# Disease associations depend on visit type: results from a visit-wide association study

**DOI:** 10.1186/s13040-019-0203-2

**Published:** 2019-07-11

**Authors:** Mary Regina Boland, Snigdha Alur-Gupta, Lisa Levine, Peter Gabriel, Graciela Gonzalez-Hernandez

**Affiliations:** 1 Department of Biostatistics, Epidemiology & Informatics, University of Pennsylvania, Philadelphia, PA 19104 USA; 2 Institute for Biomedical Informatics, University of Pennsylvania, Philadelphia, PA 19104 USA; 3 Center for Excellence in Environmental Toxicology, University of Pennsylvania, Philadelphia, PA 19104 USA; 4 Department of Biomedical and Health Informatics, Children’s Hospital of Philadelphia, 421 Blockley Hall, Philadelphia, PA 19104 USA; 5 Department of Obstetrics & Gynecology, University of Pennsylvania, 421 Blockley Hall, Philadelphia, PA 19104 USA; 6 Department of Radiology, University of Pennsylvania, Philadelphia, PA 19104 USA

## Abstract

**Introduction:**

Widespread adoption of Electronic Health Records (EHR) increased the number of reported disease association studies, or Phenome-Wide Association Studies (PheWAS). Traditional PheWAS studies ignore **visit type** (i.e., department/service conducting the visit). In this study, we investigate the role of visit type on disease association results in the first Visit-Wide Association Study or ‘VisitWAS’.

**Results:**

We studied this visit type effect on association results using EHR data from the University of Pennsylvania. Penn EHR data comes from 1,048 different departments and clinics. We analyzed differences between cancer and obstetrics/gynecologist (Ob/Gyn) visits. Some findings were expected (i.e., increase of neoplasm diagnoses among cancer visits), but others were surprising, including an increase in infectious disease conditions among those visiting the Ob/Gyn.

**Conclusion:**

We conclude that assessing visit type is important for EHR studies because different medical centers have different visit type distributions. To increase reproducibility among EHR data mining algorithms, we recommend that researchers report visit type in studies.

**Electronic supplementary material:**

The online version of this article (10.1186/s13040-019-0203-2) contains supplementary material, which is available to authorized users.

## Introduction

Widespread adoption of Electronic Health Records (EHRs) began in the United States of America (USA) following Federal guidelines passed in 2009 [1]. With the sudden increase in phenotypic data available in EHRs, many studies began to investigate the relationship between genetic variants and disease phenotypes extracted from the EHR.. These association studies are often referred to as Phenome-Wide Association Studies or PheWAS [2]. The original PheWAS studies focused on comparing gene – disease associations within EHRs linked with Biobanked genetic data [3]. These gene-disease association studies investigated hypothyroidism [4], platelet count [5], and even alcohol and nicotine risk alleles [6]. Original studies used structured disease codes and investigated genes associated with these structured codes. However, others have used terms extracted from clinical text *in lieu* of structured data and also found genes associated with certain clinical disease terms [7]. The original PheWAS studies investigate multiple Single Nucleotide Polymorphisms (SNPs) and their association with a few specific diseases extracted from the EHR, such as hypothyroidism [4].

Furthermore, consortiums such as Pediatric Network (PEDSnet), Observational Health Data Sciences and Informatics (OHDSI) and Patient Centered Outcomes Research Institute (PCORI) were developed to share EHR data and results within subsets of reporting hospitals. OHDSI consists of a network of academic medical centers throughout the world that each house their own EHR or claims data, including from different types of healthcare systems (e.g., cancer hospital, community hospital), locally. Results are then compiled from across the network and shared [8]. Individual researchers can also conduct their studies in a meta-analysis framework with individual sites sharing estimates rather then patient-level data [9].

With the expansion of the number of EHR datasets available from across the country and the world, more associations were conducted, typically between a single key phenotype (e.g., periodontal disease) and many diseases simultaneously extracted from EHRs [10]. Later work involved investigating the relationship between birth month/season and all diseases extracted from the EHR having at least 1000 patients [11]. These studies follow the framework of ‘disease association studies’ where a large number of diseases are simultaneously investigated for their relationship or association with a key exposure or other outcome of interest.

However, neither PheWAS studies nor many EHR based association studies investigate the effects of visit type on results. The importance of visit type in PheWAS studies has been discussed as future work in several studies [12, 13]. However, no available methods have been developed to investigate the effect of visit type on PheWAS studies.

The purpose of this study is to investigate the relationship between visit type and disease associations revealed from the EHR. We call this method ‘VisitWAS’ to signifiy Visit-Wide Association Study. Instead of investigating the relationship between one key disease or outcome’s relationship with other diseases in the EHR as in traditional ‘Wide Association Studies’, we are studying the effect of the visit type on the diseases in the EHR. We focus on two groups that are experience a large number of different types of pain diagnoses (i.e., ‘high pain’) groups – those visiting Ob/Gyn and those visiting a cancer clinic. We will also perform a sub-analysis on pain diagnoses to investigate pain diagnoses that are associated with Ob/Gyn visits vs. cancer visits. We chose to investigate pain diagnoses because many pain diagnoses, e.g., ‘pain in abdomen’ are vague and can mean very different things depending on the context of the code (i.e., was it a cancer visit or an Ob/Gyn visit where this code was used). Our work sheds light on the importance of visit type in PheWAS studies.

## Results

### Dataset

**Fig. 1:**
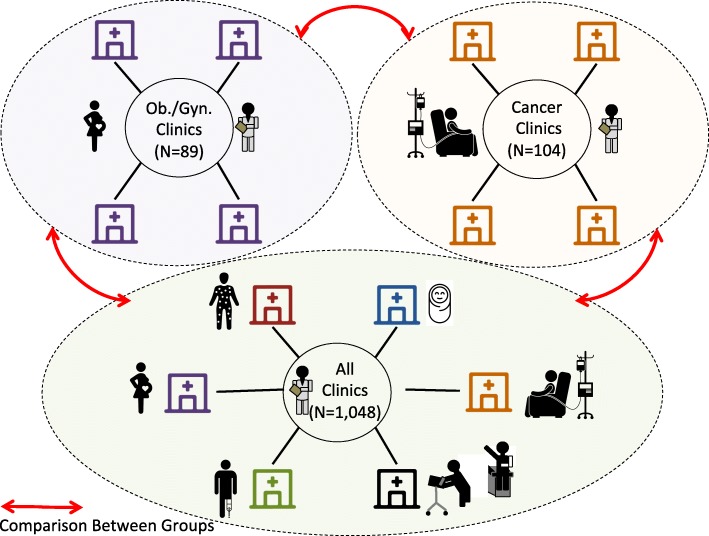
Diagram Showing Comparison Between Clinic Types: Ob./Gyn., Cancer and All. We compared 1) Ob./Gyn. Visits vs. All Visits, 2) Cancer Visits vs. All Visits and 3) Ob./Gyn. Visits vs. Cancer Visits (red lines in Figure)

This study was conducted using out-patient data obtained from the Hospital of the University of Pennsylvania (called hereafter Penn). Penn contains data from the Philadelphia Metropolitan Area. This includes outpatient data from Southern New Jersey, Philadelphia, parts of Delaware and in the Pennsylvania suburbs of Philadelphia. These data are all structured data collected during routine clinical care between the period of 2006 and 2017. We used women only to demonstrate the effect of visit type on disease associations and also to compare pain diagnoses across cohorts (Fig. 1). Our ‘all clinics’ as shown in Fig. 1 represent all clinics at UPenn where our cohort of women were treated. This includes clinics for cardiology, rheumatology, immunology to name a few. We only investigate women in our analyses because we compare those being treated Ob/Gyn visits versus cancer visits and other visits. Since Ob/Gyn visits generally are made by females it would not make sense to compare against males. In addition many diagnoses, including pain, vary by sex. The demographics of women included in this study are found in Table 1. We included women with an Ob/Gyn visit and outpatient diagnosis code information, women with a cancer visit and outpatient diagnosis code information and all women with diagnoses found in the EHR. The average age for women visiting a cancer clinic was 56 years while those visiting Ob/Gyn clinics were younger (41 years). Because women have multiple visits that can span over several years, we calculated each woman’s average age across their record and then we calculated the overall average and standard deviation shown in Table 1. For defining diagnoses, we used the International Classification of Diseases (ICD) version 9 (ICD-9) and version 10 (ICD-10). The ICD-9 and ICD-10 codes are used primarily for billing purposes to describe the conditions, symptoms, illnesses and diseases for a given patient. We collectively term ICD-9 and ICD-10 diagnoses as conditions in our study.

**Table 1:** Demographics of Women Treated at Penn

	Women with an Ob./Gyn. Visit (*N* = 233,069)	Women with a Cancer Visit (*N* = 77,967)	Women in EHR (*N* = 742,861)
Race			
White	136,355 (58.50%)	54472 (69.87%)	462,675 (62.28%)
African American	68071 (29.21%)	15729 (20.17%)	164,931 (22.20%)
Other	17,522 (7.52%)	5649 (7.25%)	88,386 (11.90%)
Asian	11,121 (4.77%)	2117 (2.72%)	27,880 (3.75%)
Mean Age	41 years	56 years	48 years
Standard Deviation Age	16.19 years	15.96 years	18.75 years
Num. of Clinics	89	104	1,048

Importantly, we did not require women have a specific diagnosis of cancer, only that they visited a cancer clinic. Likewise, we did not require that women were pregnant or had a diagnosis of a pregnancy. We only required that they had visited an Ob/Gyn clinic. Women visit Ob/Gyn clinics during all stages of their life, this includes from menarche, childbearing years, during menopause and even after menopause). A total of 1,048 departments and clinics consist within the Penn outpatient health system. Of these 89 are Ob/Gyn/reproductive endocrinology and family planning departments/clinics (8.49%) and only 104 are cancer or oncology departments/clinics (9.92%).

### Visit-PheWAS or VisitWAS

We performed a PheWAS using visit type or VisitWAS. This resulted in many disease associations that were correlated with either Ob/Gyn or cancer visits. Overall, there were a total of 7,186 conditions coded for in our dataset. Conditions include diseases (e.g., breast cancer), symptoms (e.g., lump in breast), and infections (e.g., flu) provided that there is a distinct ICD-9 or ICD-10 for that condition. In our VisitWAS study, we performed three different comparisons: 1) those treated at an Ob/Gyn clinic versus any visit (i.e., the entire population), 2) those treated at a cancer clinic versus any visit (i.e., the entire population) and 3) those treated at an Ob/Gyn clinic versus those treated at a cancer clinic. Because patients visiting the Ob/Gyn and those visiting a cancer clinic often visit frequently it made sense to compare these two groups to each other to highlight conditions that were more strongly associated with one group versus the other. We found that many conditions were associated with visit type after adjusting for multiple testing using Bonferroni correction (Additional file 1: Table S1). Out of 7,186 conditions, we found 2,150 significantly associated with an Ob/Gyn visit or 29.92% of all conditions. For cancer visits, we found that 33.58% of conditions were associated (2413/7186). These counts are based on a 0.05 significance threshold that was adjusted for the 7,186 tests we conducted. We also computed the proportion of association conditions that were pain-related along with the proportion of pain conditions associated (out of a total of 129 possible pain conditions).

**Fig. 2:**
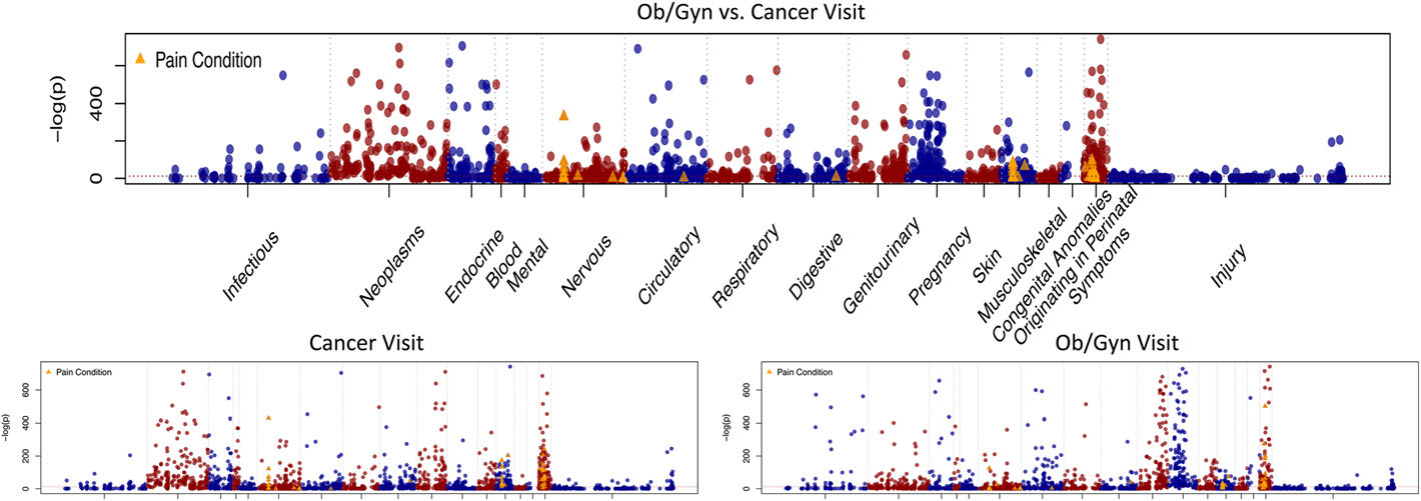
Manhattan Plots Showing Condition/Disease Associations by Visit Type. The top plot shows conditions that are either positively or negatively associated with Ob/Gyn visits when compared to cancer visits. The bottom two subplots show the significant associations when cancer visits are compared to all visits (left) and when Ob/Gyn visits are compared to all visits (right). All plots are –log(*p*-value) for the association on the y-axis and x-axis shows the ICD-9 categories. Pain conditions are denoted by orange triangles. We alternate between blue and red to highlight the different disease categories. Diagram Showing Comparison Between Clinic Types: Ob./Gyn., Cancer and All. We compared 1) Ob./Gyn. Visits vs. All Visits (lower righthand corner), 2) Cancer Visits vs. All Visits (lower lefthand corner) and 3) Ob./Gyn. Visits vs. Cancer Visits (top large figure)

**Fig. 3:**
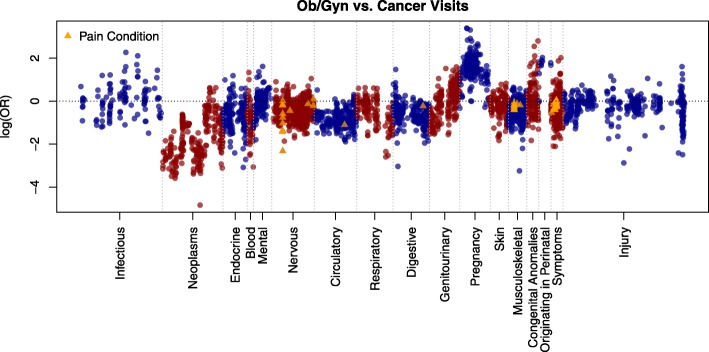
Manhattan Plot of log (Odds Ratio) of Association between Conditions from an Ob/Gyn vs. a Cancer Visit. Positive Associations (log (OR) > 0) indicate that the diagnosis is more common among those who visit an Ob/Gyn clinic. Negative Associations (log (OR) < 0) indicate that the diagnosis is more common among those who visit a Cancer clinic. The y-axis shows the log (Odds Ratio) while the x-axis shows ICD-9 condition categories. Pain conditions denoted by orange triangles. We alternate between blue and red to highlight the different disease categories. Note that more nervous system pain conditions are associated with cancer visits (log (OR) < 0 in Fig. 3

The Manhattan plots for the association between diseases and visit type are shown in Fig. 2. The Manhattan plots only show the associations for ICD-9 disease categories. We excluded ICD-10 and V codes from the plots. Many of the associations are as expected. Neoplasm diagnoses are strongly associated with cancer visits (lower left hand graph in Fig. 2). Pregnancy, genitourinary and symptom diagnoses are strongly associated with Ob/Gyn visits. Fig. 3 shows the log of the Odds Ratio for the association of diseases to ob./gyn visits vs. cancer visits, which clearly illustrates diseases associated with ob./gyn. Visits (log (OR) > 0) and those associated with cancer visits (log (OR) < 0). Table 2 shows the number of significant associations by ICD-9 disease category broken down by visit type after adjusting for multiple comparisons.

**Table 2:** Number of Associations* by Visit Type and Disease Category

Disease Category	Ob./Gyn. Visit	Cancer Visit
Infectious	35	27
Neoplasms	96	216
Endocrine	27	83
Blood	13	42
Mental	28	17
Nervous	72	84
Circulatory	95	57
Respiratory	38	33
Digestive	33	85
Genitourinary	147	99
Pregnancy	181	35
Skin	39	45
Muscle	54	100
Congenital	16	6
Originating in Perinatal	10	2
Symptoms	112	157
Injury	28	35
ICD-10 and V Codes	1126	1290

*Adjusted Using Bonferroni Correction

### Pain depends on visit type

Overall 129 pain conditions were found in our dataset. We looked at the proportion of associated conditions that were pain related and found that 43 or 2.00% (43/2150) of unique conditions associated with Ob/Gyn visits were pain related (Additional file 1: Table S2). We found that more pain conditions were associated with individuals who visited cancer clinics with 76 or 3.15% of all associations being pain related. For Ob/Gyn visits, we found that 33.33% of pain conditions were associated with Ob/Gyn visits versus 58.91% of pain conditions that were associated with cancer visits. Many of these pain conditions were general and non-specific. When we compared Ob/Gyn vs cancer visits, we found 43 significant pain associations. Only one of these pain conditions was positively associated with Ob/Gyn visits while the remaining 42 pain conditions were associated with cancer visits (or negatively associated with Ob/Gyn visits) as shown in Additional file 1: Table S2. The type of pain unique to Ob/Gyn visits was ‘Pelvis and Perineal pain’ (OR = 2.086). The pain diagnoses that were most associated with cancer visits, i.e., most negatively associated with Ob/Gyn visits (OR < < 1), include ‘neoplasm related pain acute and chronic’ (OR = 0.108 and OR = 0.099 depending slightly on ICD-9 or ICD-10). Also Acute post-thoracotomy pain was heavily associated with cancer visits (OR = 0.243) and Other chronic postoperative pain (OR = 0.359). ‘Chronic pain syndrome’ was also heavily associated with cancer visits with OR = 0.47. We kept the ICD-9 and ICD-10 diagnoses separate because many of the codes in these terminologies differ in terms of their granularity [14] and granularity can effect the coverage of the code set resulting in mapping difficulties [15–17]. Importantly, codes that were exact matches between the two terminologies were both associated and the associations are similar in direction and size.

## Discussion

Our study found that visit type significantly affects the kinds of associations revealed by high-throughput EHR data mining algorithms or PheWAS studies.

### Implications of visit type for PheWAS studies

While the importance of visit type in PheWAS studies has been discussed as future work for the past few years [12, 13], no available methods for quantifying the effects of visit type on PheWAS studies have been conducted. We leveraged Penn’s integrated health system containing 1,048 departments/clinics to stratify our cohorts by patients who have been treated by cancer clinics, ob./gyn. Clinics and all clinics to determine the result effects on diagnosis code associations. Some of our findings were expected, including an increase in neoplasm codes for those treated at a cancer clinic, and increases in genitourinary and pregnancy codes for those treated at an ob./gyn. Clinic. However, some conditions – for example, the observed increase in infectious disease condition codes among those treated at an ob./gyn. Clinic vs. those treated at a cancer clinic (Fig. 3) may be surprising initially. Cancer disease treatment and progression have been linked with increased susceptibility to infectious diseases [18]. Furthermore, many chemotherapy agents affect the immune system [19]. Therefore, one might expect those at a cancer clinic to have an increased risk of infectious diseases. However, our visit-PheWAS revealed that they were at decreased risk of infectious diseases when compared with the ob./gyn. Group. Women who visit the ob./gyn. Both inside and outside of the pregnancy state are tested for a host of conditions that affect the immune system, including vaginal infections, sexually transmitted infections (STIs), and urinary tract infections (UTIs). Also women are also routinely tested for cervical cancer via the Papanicolaou (PAP) smear test [20] that indicates damage to the cervix due to Human Papilloma Virus (HPV). These factors could result in an increase of infectious related diagnoses among the ob./gyn. Cohort when compared with the cancer cohort (Fig. 3). Therefore, taking visit type into account when performing EHR-based PheWAS is more important or else the results of the PheWAS may be skewed towards certain diseases, infections and symptoms.

### Comparing diagnoses within two high-pain groups: Cancer and Ob/Gyn

We compared two traditionally high-pain cohorts: ob./gyn. and cancer to determine what types of pain distinguish these two groups from one another (Additional file 1: Table S2). We found that one pain diagnosis is specific to Ob/Gyn visits, namely pelvis and perineal pain. On the other hand many different types of pain are associated with cancer visits, including chronic pain, pos-operative pain codes and other specific body location-related pain (Additional file 1: Table S2). It is important to compare the two high-pain groups to each other (Fig. 1) rather then comparing against all clinics because 33.33% of all 129 pain codes in our dataset were significantly associated with ob./gyn. Visits and close to 60% or 58.91% of all pain conditions were associated with cancer visits (Table 2). Therefore, patients visiting either Ob/Gyn clinics or cancer clinics tend to experience a high degree of pain related conditions.

### Limitations and future work

A limitation of our work includes our use of structured data alone. Including text mining of the clinical notes to identify further sources of pain not included in the structured billing codes would enhance our analysis. We kept the analysis at the individual code level without mapping between terminologies because of the differences in granularity between ICD-9 and ICD-10. Future work could leverage ontologies to measure the effects on codes at different granularity levels.

## Conclusion

In conclusion, this PheWAS of visit type illustrates the importance of considering visit type prevalence within an EHR dataset. This has important implications in terms of reproducibility. For example, if a study is conducted in an EHR system containing more cancer clinics then the cancer visit type will dominate in that setting. This will result in certain associations that may not reproduce when the study is rerun at a community hospital where Ob/Gyn visits dominate. With regards to pain, there are 42 distinct pain conditions associated with those who visit cancer clinics while 1 pain condition – pelvic and perineal pain was associated with those who visit Ob/Gyn clinics. This demonstrates the high degree of pain burden experienced by those seeking cancer treatment and could be important for reproducibility of studies across sites depending on whether cancer patients are included, or excluded from the analysis. This study is the first ‘VisitWAS’ investigating the role of Visit type on condition associations and therefore is important to include visit type in all PheWAS studies.

## Materials and methods

### Dataset

We obtained data for patients treated at the Hospital of the University of Pennsylvania (called hereafter Penn). We include in our sample only females treated at Penn, we chose only women because we wanted to explore pain diagnoses among those treated at Ob/Gyn clinics. Since men are not typically treated at Ob/Gyn clinics, we performed our ‘VisitWAS’ only among women to meaningfully compare groups. Using clinic location information, we grouped females into those treated at an Ob/Gyn clinic versus those treated at a cancer clinic versus any clinic type. We defined an Ob/Gyn clinic by using the EPIC department id categories and manual review of the departments to ensure that all female reproductive health clinics were included. We also defined a cancer/oncology department clinic by manually curating EPIC department IDs. Reproductive oncology clinics were labeled as both cancer and Ob/Gyn clinics.

### PheWAS on visit type or VisitWAS

We performed association analysis using Fisher’s exact test for the association between outpatient diagnosis codes and certain visit types, following the PheWAS framework [2]. We investigated the association between each diagnosis code and visit type provided that the diagnosis code appeared in at least 50 patients at Penn. We chose Fisher’s exact test because it is robust for small sample sizes. We restricted our analysis to only include those diagnoses with at least 50 patients overall. However, the sample size for a given disease at a particular visit type could be less then 5 patients and therefore Fisher’s exact test is more appropriate than Chi-square. We performed association analysis at the code level for associations between a given code and either cancer visits or Ob/Gyn visits. We used both ICD-9 and ICD-10 codes and did not map between them. We illustrated our results in a Manhattan plot format only for the ICD-9 disease categories, excluding the ICD-10 and V codes from the plots. We then adjusted for multiple comparisons using the Bonferroni method that controls the Family-Wise Error Rate. Because 7,186 diagnoses (including both ICD-9 and ICD-10) occurred in at least 50 patients, we performed a total of 7,186 tests (*p*-value for significance = 0.05/7186).

### Pain diagnosis sub-analysis

We were interested in pain diagnoses and how they vary by visit type. Therefore, we identified all pain diagnoses from the 7,186 diagnoses at Penn occuring in at least 50 patients. A total of 129 diagnoses were pain related out of 7,186 or 1.80%. This allowed us to compare not only diagnosis associations that varied by visit type (section 2.2), but also pain diagnosis associations and how those varied by visit type.

### Comparison Group in Association Analysis

To show the effect of visit type on disease associations. We compared 3 groups: 1) those treated at an Ob/Gyn clinic versus any visit, 2) those treated at a cancer clinic versus any visit and 3) those treated at an Ob/Gyn clinic versus a cancer clinic. Patients visiting the Ob/Gyn and also cancer clinics tend to visit frequently. Therefore, we devised the third group – Ob/Gyn clinic vs. cancer clinic to adjust for the high number of diagnoses within these two patient groups. Associations within this third group represent associations that are unique to either cancer or Ob/Gyn.

## Additional file

Additional file 1: **Table S1.** Number of Associated Conditions by Visit Type (*N* = 7186). **Table S2.** Pain Conditions Associated with Ob/Gyn. vs. Cancer Visits. (DOCX 44 kb)

## Data Availability

All data are housed at the University of Pennsylvania and contain patient health information; therefore, we cannot release these data publicly. We provide details in this manuscript and in the supplemental regarding our methods and are welcome to answer any questions that researchers may have regarding our methods/approach.

## References

[1] Blumenthal D (2009). Stimulating the adoption of health information technology. N Engl J Med.

[2] Denny JC, Ritchie MD, Basford MA, Pulley JM, Bastarache L, Brown-Gentry K, Wang D, Masys DR, Roden DM, Crawford DC (2010). PheWAS: demonstrating the feasibility of a phenome-wide scan to discover gene–disease associations. Bioinformatics.

[3] Denny JC, Bastarache L, Ritchie MD, Carroll RJ, Zink R, Mosley JD, Field JR, Pulley JM, Ramirez AH, Bowton E (2013). Systematic comparison of phenome-wide association study of electronic medical record data and genome-wide association study data. Nat Biotechnol.

[4] Denny JC, Crawford DC, Ritchie MD, Bielinski SJ, Basford MA, Bradford Y, Chai HS, Bastarache L, Zuvich R, Peissig P (2011). Variants near FOXE1 are associated with hypothyroidism and other thyroid conditions: using electronic medical records for genome-and phenome-wide studies. Am J Hum Genet.

[5] Shameer K, Denny JC, Ding K, Jouni H, Crosslin DR, De Andrade M, Chute CG, Peissig P, Pacheco JA, Li R (2014). A genome-and phenome-wide association study to identify genetic variants influencing platelet count and volume and their pleiotropic effects. Hum Genet.

[6] Polimanti R, Kranzler HR, Gelernter J (2016). Phenome-wide association study for alcohol and nicotine risk alleles in 26394 women. Neuropsychopharmacology.

[7] Hebbring SJ, Rastegar-Mojarad M, Ye Z, Mayer J, Jacobson C, Lin S (2015). Application of clinical text data for phenome-wide association studies (PheWASs). Bioinformatics.

[8] Hripcsak G, Ryan PB, Duke JD, Shah NH, Park RW, Huser V, Suchard MA, Schuemie MJ, DeFalco FJ, Perotte A (2016). Characterizing treatment pathways at scale using the OHDSI network. Proc Natl Acad Sci.

[9] Boland MR, Parhi P, Li L, Miotto R, Carroll R, Iqbal U, Nguyen P-A, Schuemie M, You SC, Smith D (2018). Uncovering exposures responsible for birth season – disease effects: a global study. J Am Med Inform Assoc.

[10] Boland MR, Hripcsak G, Albers DJ, Wei Y, Wilcox AB, Wei J, Li J, Lin S, Breene M, Myers R (2013). Discovering medical conditions associated with periodontitis using linked electronic health records. J Clin Periodontol.

[11] Boland MR, Shahn Z, Madigan D, Hripcsak G, Tatonetti NP (2015). Birth month affects lifetime disease risk: a phenome-wide method. J Am Med Inform Assoc.

[12] Wright A, Chen ES, Maloney FL (2010). An automated technique for identifying associations between medications, laboratory results and problems. J Biomed Inform.

[13] Boland MR, Hripcsak G, Shen Y, Chung WK, Weng C (2013). Defining a comprehensive verotype using electronic health records for personalized medicine. J Am Med Inform Assoc.

[14] Manchikanti L, Kaye AD, Singh V, Boswell MV (2015). The tragedy of the implementation of ICD-10-CM as ICD-10: is the cart before the horse or is there a tragic paradox of misinformation and ignorance. Pain physician.

[15] Wei W-Q, Bastarache LA, Carroll RJ, Marlo JE, Osterman TJ, Gamazon ER, Cox NJ, Roden DM, Denny JC (2017). Evaluating phecodes, clinical classification software, and ICD-9-CM codes for phenome-wide association studies in the electronic health record. PLoS One.

[16] Columbo JA, Kang R, Trooboff SW, Jahn KS, Martinez CJ, Moore KO, Austin AM, Morden NE, Brooks CG, Skinner JS (2018). Validating publicly available crosswalks for translating ICD-9 to ICD-10 diagnosis codes for cardiovascular outcomes research. Circ Cardiovasc Qual Outcomes.

[17] Utter Garth H., Cox Ginger L., Atolagbe Oluseun O., Owens Pamela L., Romano Patrick S. (2018). Conversion of the Agency for Healthcare Research and Quality's Quality Indicators from ICD-9-CM to ICD-10-CM/PCS: The Process, Results, and Implications for Users. Health Services Research.

[18] Reiche EMV, Nunes SOV, Morimoto HK (2004). Stress, depression, the immune system, and cancer. Lancet Oncol.

[19] Zitvogel L, Kepp O, Kroemer G (2011). Immune parameters affecting the efficacy of chemotherapeutic regimens. Nat Rev Clin Oncol.

[20] Control CfD (2013). Prevention: cervical cancer screening among women aged 18-30 years-United States 2000-2010. MMWR Morb Mortal Wkly Rep.

