# Supplementary material for: Disease associations depend on visit type: results from a visit-wide association study
Source: BioData Min. 2019 Jul 11;12:15. doi: 10.1186/s13040-019-0203-2 (PMC6625053; doi:10.1186/s13040-019-0203-2)
Supplement: Supplementary file 1 — Table S1. Number of Associated Conditions by Visit Type (N = 7186). Table S2. Pain Conditions Associated with Ob/Gyn. vs. Cancer Visits. (DOCX 44 kb) [file 13040_2019_203_MOESM1_ESM.docx]

**Supplemental Information for:**

**Boland MR, Alur-Gupta S, Levine L, Gabriel P, Gonzalez-Hernandez G.**

Importance of Visit Type in Understanding Results from Phenome-Wide Association Studies: Results from a Visit-WAS

**Table S1.**Number of Associated Conditions by Visit Type (N=7186)

|  | **Ob./GynVisit vs. Any Visit** | **Cancer Visit vs. Any Visit** | **Ob./Gyn Visit vs. Cancer Visit** |
| --- | --- | --- | --- |
| Num. Associated Conditions * | 2150 (29.92%) | 2413 (33.58%) | 2598 (36.15%) |
| Num. Pain-Related Associated Conditions | 43 | 76 | 43 |
| Prop. Of Associated Conditions that are Pain-Related | 2.00% (43/2150) | 3.15% (76/2413) | 1.66% (43/2598) |
| Prop. Of Pain Conditions that are Associated | 33.33% (43/129) | 58.91% (76/129) | 33.33% (43/129) |

*Adjusted Using Bonferroni Correction

**Table S2.**Pain Conditions Associated with Ob/Gyn. vs. Cancer Visits

| **Code** | **Pain Condition** | **OR** |
| --- | --- | --- |
| R10.2 | Pelvis and Perineal Pain | 2.086 |
| 719.47 | Pain in joint, ankle and foot | 0.849* |
| M54.5 | Low back pain | 0.834 |
| 789.01 | Abdominal pain, right upper quadrant | 0.833 |
| 729.5 | Pain in limb | 0.827 |
| 719.46 | Pain in joint, lower leg | 0.817 |
| 789.06 | Abdominal pain, epigastric | 0.806 |
| M25.562 | Pain in left knee | 0.773 |
| 786.5 | Chest pain, unspecified | 0.768 |
| 789.07 | Abdominal pain, generalized | 0.764 |
| 719.41 | Pain in joint, shoulder region | 0.756 |
| 719.45 | Pain in joint, pelvic region and thigh | 0.746 |
| 786.59 | Other chest pain | 0.730 |
| R07.89 | Other chest pain | 0.715 |
| M25.561 | Paint in right knee generalized | 0.714 |
| R10.84 | Abdominal pain | 0.698 |
| R07.2 | Precordial pain | 0.696 |
| M25.552 | Pain in left hip | 0.689 |
| 719.4 | Pain in joint, site unspecified | 0.686 |
| M25.512 | Pain in left shoulder | 0.675 |
| M25.511 | Pain in right shoulder | 0.673 |
| 719.49 | Pain in joint, multiple sites | 0.672 |
| M25.551 | Pain in right hip | 0.671 |
| 786.52 | Painful respiration | 0.669 |
| 719.44 | Pain in joint, hand | 0.668 |
| 786.51 | Precordial pain | 0.663 |
| M25.569 | Pain in unspecified knee | 0.645 |
| 350.2 | Atypical face pain | 0.639 |
| M25.50 | Pain in unspecified joint | 0.635 |
| R52 | Pain, unspecified | 0.601 |
| M79.605 | Pain in left leg | 0.597 |
| 780.96 | Generalized pain | 0.581 |
| M79.601 | Pain in right arm | 0.567 |
| 338.29 | Other chronic pain | 0.554 |
| M25.519 | Pain in unspecified shoulder | 0.551 |
| M79.643 | Pain in unspecified hand | 0.519 |
| G89.29 | Other chronic pain | 0.493 |
| G89.4 | Chronic pain syndrome | 0.474 |
| 338.4 | Chronic pain syndrome | 0.471 |
| 338.28 | Other chronic postoperative pain | 0.359 |
| 338.12 | Acute post-thoracotomy pain | 0.243 |
| G89.3 | Neoplasm related pain (acute)(chronic) | 0.108 |
| 338.3 | Neoplasm related pain (acute)(chronic) | 0.099 |

**** OR’s <1 indicate that the pain code is associated with cancer visits***
